# Prediction of response to bacillus Calmette-Guérin treatment in non-muscle invasive bladder cancer patients through interleukin-6 and interleukin-10 ratio

**DOI:** 10.3892/etm.2012.634

**Published:** 2012-07-05

**Authors:** TOMMASO CAI, GABRIELLA NESI, SANDRA MAZZOLI, FRANCESCA MEACCI, GALLIANO TINACCI, LORENZO GIUSEPPE LUCIANI, VINCENZO FICARRA, GIANNI MALOSSINI, RICCARDO BARTOLETTI

**Affiliations:** 1 Department of Urology, Santa Chiara Hospital, Trento;; 2 Division of Pathological Anatomy, Department of Critical Care Medicine and Surgery, University of Florence, Florence;; 3 STD_s_ Center and; 4 Department of Pathology, Santa Maria Annunziata Hospital, Florence;; 5 Department of Urology, University of Padua, Padua;; 6 Department of Urology, University of Florence, Florence, Italy

**Keywords:** interleukin-6, interleukin-10, bacillus Calmette-Guérin, bladder cancer, urinary bladder neoplasms, urothelial cancer, urinary cytology, recurrence

## Abstract

This study aimed to evaluate whether the interleukin-6 (IL-6) and interleukin-10 (IL-10) ratio (IL-6/IL-10) can be used as a prognostic marker of recurrence following bacillus Calmette-Guérin (BCG) therapy in patients with high-risk non-muscle invasive bladder cancer (NMIBC). One hundred and twenty-one consecutive urological patients (72 affected by high-risk NMIBC and 49 controls) were selected for this prospective study. Urine samples for dipstick and interleukin analyses were collected from each subject before surgery. All patients underwent transurethral resection of bladder tumours (TUR-BT), followed by six weekly BCG instillations. IL-6 and IL-10 concentrations in urine were determined by solid phase ELISA Quantikine IL-6 and IL-10 Immunoassay. Patients with NMIBC were stratified in accordance with IL-6/IL-10: group A ≤0.09 and group B >0.10. The main outcome measures were time to first recurrence and recurrence rate following BCG therapy. At enrolment, IL-6/IL-10 was not statistically different between patients and controls (p=0.763, degrees of freedom (df)=1, F-test result (F)=0.092). Of the 72 patients with NMIBC, 38 (52.7%) had an IL-6/IL-10 of ≤0.09 (group A), while 34 (47.3%) had an IL-6/IL-10 of >0.10 (group B). A significant difference between IL-6/IL-10 and status at follow-up was found (p=0.016, df=1, χ^2^=5.800). The Kaplan-Meier curves demonstrated that group B patients had a significantly higher probability of being recurrence-free than group A patients [p=0.003; recurrence rate (RR)=3.1]. At multivariate analysis, IL-6/IL-10 (p<0.003) and the number of lesions (p<0.001) were identified as independent predictors of BCG response probability. In conclusion, this study highlights the feasible role of IL-6/IL-10 in predicting recurrence following BCG therapy in high-risk NMIBC.

## Introduction

Intravesical bacillus Calmette-Guérin (BCG) immunotherapy is considered more effective than intravesical chemotherapy and is advocated as the first treatment choice in patients with carcinoma *in situ* or with pT1G3 (1). However, not all patients benefit from intravesical BCG (2). In fact, 30–50% of patients undergoing intravesical BCG treatment have recurring tumours following therapy (3–5), with common side-effects that can be life threatening (6). Patients refractory to BCG therapy are subjected to salvage cystectomy as standard treatment (2), though may show a poor prognosis (7). Therefore, the patient failing BCG therapy is a challenge to the urologist and predictors of BCG response should be investigated (2). Although several authors have proposed molecular markers to predict the clinical outcome of patients with non-muscle invasive bladder cancer (NMIBC) following treatment with intravesical BCG (8–9), no marker has yet proven to be valid in everyday clinical practice (2). Zhang *et al* demonstrated the induction of cytokine production by BCG, particularly interleukin-6 (IL-6) (10). Recently, Luo *et al* showed the inhibitory role of interleukin-10 (IL-10) in BCG-induced macrophage cytotoxicity, suggesting that the blockage of IL-10 may potentially enhance the effect of BCG in the treatment of bladder cancer patients (11). Chen *et al* demonstrated that BCG increases IL-6 messenger RNA and protein in a time-and dose-dependent manner via an immediate early pathway (12). Furthermore, we highlighted the feasible role of IL-6 and IL-10 ratio (IL-6/IL-10) as a prognostic marker of recurrence in patients affected by NMIBC (9). Urinary interleukins could consequently be used as prognosticators to better assess the outcome of NMIBC patients following BCG therapy. The present study aimed to evaluate the role of IL-6/IL-10 in predicting patient response to BCG therapy.

## Materials and methods

### 

#### Study design

To evaluate the prognostic role of urinary IL-6/IL-10, all consecutive patients who had undergone transurethral resection of bladder tumours (TUR-BT) at the same urological unit (Department of Urology, University of Florence, Florence, Italy) between January 2005 and May 2006 were selected for this prospective study. As controls, 49 subjects were enrolled from among patients attending the clinic in the same period for other non-malignant urological diseases. Since this study was designed to establish the role of IL-6/IL-10 as a predictor of the response to BCG and not as a diagnostic tool, patients with other urological malignant diseases were excluded. Outcome measures were time to first recurrence following treatment and recurrence rate. Disease progression was not included among the main outcomes due to the small number of patients who experienced stage or grade progression, though these data are reported in Results. Response to BCG was confirmed by negative urinary cytology and cystoscopy after 6 months’ follow-up (13). According to Herr and Dalbagni, patients were defined as BCG-refractory if the recurrence occurred within 6 months of starting BCG treatment (14). Urine samples for the urine dipstick test and interleukin analyses were collected before TUR-BT and 6 subsequent weekly BCG instillations. Depending on laboratory results, all patients enrolled in the study were divided into 2 groups: group A with IL-6/IL-10 ≤0.09 and group B with IL-6/IL-10 >0.10. The controls constituted group C. Follow-up data were compared and analysed. Patients with no further information available at follow-up were excluded from the study. The local research ethics committee approved the study and written informed consent was obtained from all patients.

#### Eligibility for the study

Inclusion criteria were the presence of cystoscopically demonstrated bladder tumours, normal pre-operative blood tests and compliance with instillation and follow-up schedules. We enrolled only patients affected by high-risk NMIBC, according to the European Association of Urology Guidelines criteria (13). Exclusion criteria were evidence of locally infiltrative or metastatic bladder tumours (pT ≥2), presence of upper urinary tract tumours, lesions that could not be completely removed transurethrally and presence of other neoplastic, lower urinary tract or major concomitant diseases. Patients who had undergone surgery for benign prostatic obstruction at the same time as surgical treatment for NMIBC were also excluded, along with patients with histologically documented urothelial carcinoma *in situ*. Only those with a tumour recurrence of the same stage and grade of the initial tumour at diagnosis were enrolled. We enrolled only patients with recurrent urothelial cancer in order to obtain a homogeneous group for analysis. Patients with histologically confirmed pT1G3 were excluded, since management decisions in these patients are critical (15). A recurrent bladder tumour within 3 months of receiving TUR-BT was also a criterion for exclusion. Patients who had previously received intravesical treatment were included, provided this treatment had been administered >6 months before TUR-BT. No patient underwent early single intravesical instillation, as suggested by Cai *et al* (16).

#### Treatment plan

At least 21 days after TUR-BT, all patients underwent weekly BCG instillations for 6 weeks (5×10^8^ colony-forming units in 50 ml saline; OncoTice^®^ BCG, Organon USA Inc.). In accordance with Lamm *et al* (17), boosters of BCG were given at 3, 6, 12, 18, 24, 30 and 36 months. This protocol schedule is the current standard treatment for NMIBC (13). All patients were instructed to retain the drug solution in the bladder for 1 h.

#### Sample collection and storage

Urine samples were collected from each subject by using a clean catch. A spontaneous morning void urine sample was collected by the patients before TUR-BT and by the controls on enrolment. The urine sample was collected from each subject in order to obtain a basal IL-6 and IL-10 mean value. All samples were stored in aliquots at −80°C until analysis after testing for the presence of inflammatory cells or bacteriuria by using urine dipstick test for nitrites and leukocyte esterase (Multistik Pro^®^), as shown by Cai *et al* (18–19). A positive test result was indicative of urinary tract infection.

#### Interleukin measurements

At the time of the analysis, urine aliquots were defrosted and centrifuged for 10 min at 800 rpm and the urinary IL-6 and IL-10 levels were measured in the supernatants. Natural human-produced IL-6 and IL-10 concentrations were determined in the urine of all patients and controls by solid phase ELISA Quantikine IL-6 Immunoassay and IL-10 Immunoassay, respectively (R&D Systems, Minneapolis, MN, USA). All samples were measured in duplicate in accordance with the manufacturer’s recommendations (www.rndsystems.com/productdetailobjectnameQuantikine.aspx). The median minimal detectable dose of the IL-6 assay was 0.70 pg/ml and 3.9 pg/ml for IL-10. Values were considered normal if under the lowest IL-6 and IL-10 standards, 3.12 pg/ml and 7.8 pg/ml, respectively. All samples were tested in duplicate by using independent analysis to avoid errors.

#### IL-6/IL-10

The IL-6 to IL-10 ratio was calculated as a fraction between urinary IL-6 and IL-10 values, expressed as a number or fraction ranging from 0 to 1. The cut-off value was determined according to Cai *et al* (9).

#### Histopathology

Tumour stage and grade were classified following the 1998 WHO classification and the 2002 TNM classification of malignant tumours, as defined by the International Union Against Cancer. A single genito-urinary pathologist performed the pathological evaluation.

#### Patient follow-up

Each patient underwent urinary cytology, ultrasonography and cystoscopy every 3 months for the first 12 months following TUR and then every 6 months. Recurrence was determined by papillary formations protruding into the bladder lumen and detected on cystoscopy, or by the presence of neoplastic tissue diagnosed following vesical biopsy. Every recurrence was pathologically confirmed. Time to the first recurrence was defined as the time from randomisation to the date of the first recurrence. Progression was defined as an increase in tumour stage or grade (13).

#### Statistical analysis

As the null hypothesis, we assumed that there was no difference among the groups in terms of recurrence-free time or recurrence rate and that the IL-6/IL-10 had no impact on predicting the response to BCG therapy. The Fisher’s exact test and the Chi-square χ^2^ test were used to assess the significance of all parameters, and p<0.05 was considered to indicate a statistically significant difference. Kaplan-Meier survival curves and the log-rank test were also used to evaluate survival in NMIBC cases. The 95% confidence intervals (CI) were calculated for the probability of survival for Kaplan-Meier estimates. The Mann-Whitney test was also used to compare different parameter means. Univariate and multivariate relative risk was calculated by using Cox’s proportional hazards regression. Statistical analysis was performed using StatPlus for Apple Macintosh (Analyst Soft, 2001–2009).

## Results

### 

Among 196 consecutive patients undergoing TUR-BT, 81 were enrolled, of which 72 were found to be eligible for the present study. This trial ultimately included 72 patients and 49 controls. Patient characteristics, clinical and laboratory data are described in Table I.

#### Results at enrolment

**Figure 1:**
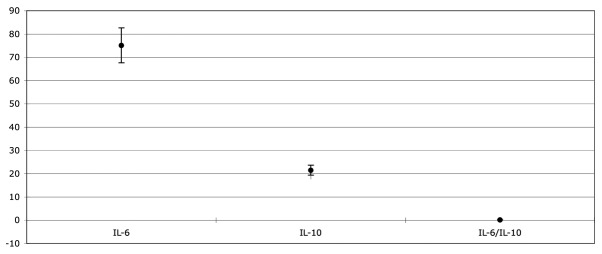
Box-plot showing the mean values of interleukin-6, interleukin-10 as well as interleukin-6 and interleukin-10 ratio among all enrolled patients.

No patient tested positive in the urine dipstick test. IL-6 and IL-10 were 74.13 pg/ml (22.84 ± SD) and 196.28 pg/ml (36.59 ± SD), respectively, among the patients, while IL-6 and IL-10 were 3.11 pg/ml (1.13 ± SD) and 5.78 pg/ml (2.17 ± SD), respectively, in the control group. The IL-6/IL-10 mean value was 0.13 (0.13 ± SD) among the patients (Fig. 1). At the baseline, 38 out of 72 (52.7%) patients showed an IL-6/IL-10 of ≤0.09 while 34 (47.3%) showed an IL-6/IL-10 of >0.10. The IL-6/IL-10 ratio was not calculated in the control group. All patients were then split into 2 groups: 38 patients to group A (IL-6/IL-10 ≤0.09) and 34 to group B (IL-6/IL-10 >0.10). In group A the IL-6/IL-10 mean value was 0.05 (0.01 ± SD) while in group B it was 0.17 (0.11 ± SD).

#### Follow-up results

All patients correctly underwent BCG intravesical therapy. Nineteen patients had mild adverse effects, which did not require treatment suspension and which were cured with antibiotic and anti-inflammatory drugs. At the end of the follow-up period (mean, 53.7 months; ranging from 49 to 58 months), all patients were alive, 39 (54.1%) without recurrence, while 33 (45.9%) had at least one recurrence. Three patients showed progression in stage and/or grade: 2 pTaG2 to pT1G3 (time to progression, 8 months) and 1 pTaG1 to pT1G3.

### Results according to the groups

*Group A (IL-6/IL-10 ≤0.09).* Among patients with IL-6/ IL-10 ≤0.09, 23 out of 38 (60.5%) showed at least one recurrence (mean, 6.2 months; ranging from 3 to 16 months), while 15 (39.5%) experienced no recurrence (mean, 13.1 months; ranging from 11 to 16 months). Among the patients with at least one recurrence at follow-up, 2 patients showed progression in stage and grade (2 pTaG2 to pT1G3).

*Group B (IL-6/IL-10 >0.10).* Among patients with IL-6/ IL-10 >0.10, 10 out of 34 (29.4%) showed at least one recurrence (mean, 12.3 months; ranging from 10 to 14 months) while 24 (70.6%) had no recurrence (mean, 16.1 months’ ranging 11 from to 17 months). Among the patients with at least one recurrence at follow-up, one patient showed grade progression (1 pT1G1 to pT1G3). All follow-up data pertaining to the groups are detailed in Table II.

#### Statistical difference between IL-6/IL-10, other clinical or pathological factors and follow-up results

**Figure 2:**
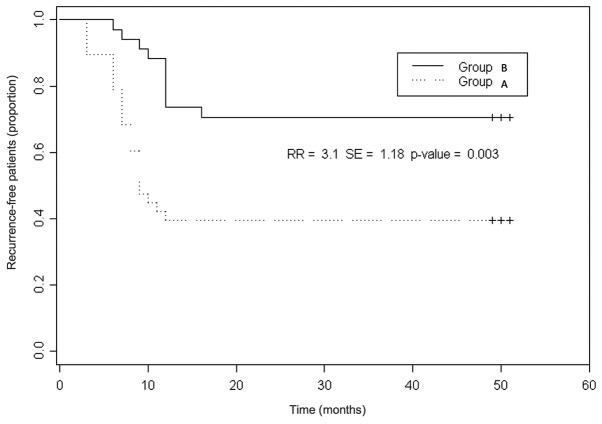
Kaplan-Meier curve analysis performed in order to calculate the probability of being recurrence-free between the 2 groups. RR, relative risk; SE, standard error.

No correlation between anamnestic or pathological factors and the IL-6/IL-10 value was reported. A statistically significant difference between the IL-6/IL-10 value and the response to BCG therapy (as status at follow-up) was found (p=0.016, df=1, χ^2^=5.800). The Kaplan-Meier analysis demonstrated a significant difference in terms of response to BCG between the 2 groups [p=0.003; recurrence rate (RR)=3.1; standard error (SE)=1.18] (Fig. 2).

#### Univariate and multivariate analysis

No clinical or anamnestic parameter had any significant impact on BCG response, as shown by univariate analysis. At multivariate analysis, the IL-6/IL-10 value (p=0.002) [IL-6/IL-10 value; hazard ratio (HR), 4.09; 95% CI, 2.59–6.28] and number of lesions (p=0.03) (3 lesions; HR, 3.31; 95% CI, 1.38–3.35) were identified as independent prognostic factors of BCG response probability (Table III).

## Discussion

In his review on the management of BCG failures in NMIBC patients, Witjes stated that the response to BCG therapy cannot be accurately predicted on an individual basis (2). In previous years, however, several molecular markers, such as urinary cytokines (20), p53 and pRb expression (6) and loss of heterozygosis on INF-α locus (9), have been proposed as prognosticators of BCG response, even if their clinical usefulness remains to be proven (2). Cai *et al* recently demonstrated that IL-6/IL-10 predicts the risk of recurrence in NMIBC patients (9). In the present study, we have shown that IL-6/IL-10 is an independent prognostic factor of BCG response probability. The rationale of this approach is based on the fact that IL-6 and IL-10 are cytokines overexpressed in bladder carcinoma, and with a role in the interaction between cancer cells and the evironment (21). IL-6 is a multifunctional cytokine produced by cells in response to several inflammatory conditions (22) and has been recognised as a growth factor for the final maturation of B cells and the differentiation of T cells (23). IL-6 is crucial in supporting systemic host response to tissue injury (24). Mulé *et al* described the anti-tumour effect of IL-6 on lung cancer in mice, confirming that the induction of host immunological response is central in the reaction to cancer (25). Moreover, several studies have demonstrated that IL-10 has the physiological role of downregulating cell-mediated immunity, resulting in the improvement of tumour immune escape (26). Nadler *et al* reported that IL-10 is an important modulator of immune-mediated events *in vivo* and suggested that efforts to downmodulate this inhibitory cytokine may be of therapeutic value (27). The theory that IL-6/IL-10 is a good prognostic marker for use in clinical practice was based on the following reasons: i) high levels of IL-10, with consequent decrease in IL-6/IL-10, indicate tumour immunosuppression, eliminating the ability of immunocompetent cells to respond to the tumour (26); ii) IL-10 is known to be a promoter of immune dysregulation, with the enhancement of Th2 cells (27). Indeed, patients with bladder cancer appear to develop a Th2 dominant status with a deficient-type immune response due to an increase in IL-10 levels (27). This immune dysregulation should be proved by altered IL-6/IL-10; iii) the decrease in IL-6 levels and increase in IL-10 levels are evidence of an altered host immune response to the tumour, confirmed by cell activation due to BCG (26). The release of immunosuppressive IL-10 during BCG therapy reduces inflammation and anticancer response (28). In addition, IL-6 and IL-10 are promoters of two different immune response pathways, i.e. Th1 and Th2, and allow the evaluation of host immunological response to the tumour (26,29). The present study, even though conducive for a better understanding of the correlation between urothelial bladder cancer cells and the host immune system, had a number of limitations that should be taken into account. First of all, the number of the patients enrolled was small, even if a statistical significance was obtained. Secondly, a group of patients undergoing alternative intravesical therapies, i.e. mitomycin or epirubicin, was lacking. Future studies are mandatory to confirm the present findings.

The current study highlights the role of IL-6/IL-10 in predicting the response to BCG therapy in NMIBC patients. We put forward the feasibility and usefulness of IL-6/IL-10 as a clinical marker, in association with traditional clinico-pathological factors, in planning a more appropriate follow-up schedule for NMIBC patients.

## Figures and Tables

**Table I:** Summary of clinical and pathological patient and control data.

A, Patient and control characteristics			

No. of patients	72		
No. of controls	49		
Median patient age (range)	71.3 (52–79)		
Median control age (range)	68.7 (57–75)		
Gender (patients)			
Male	70 (97.2)		
Female	2 (2.8)		
Gender (controls)			
Male	49 (100.0)		
Female	0 (0.0)		

B, Patient anamnestic, pathological and clinical data			

Number of recurrences/year			
1		8 (11.1)	
2		15 (20.9)	
≥3		49 (68.0)	
Number of lesions			
1		11 (15.2)	
2		25 (34.7)	
≥3		36 (50.1)	
Diameter of lesion (if multiple, diameter of the largest)			
<3 cm		44 (61.1)	
≥3 cm		28 (38.9)	
Stage			
pTa		49 (68.0)	
pT1		23 (32.0)	
Grade			
G1		8 (11.1)	
G2		26 (36.2)	
G3		38 (52.7)	
Previous intravesical therapy			
No treatment		11 (15.2)	
BCG		25 (34.7)	
Chemotherapy^a^		36 (50.1)	

C, Control clinical data			

Urological diseases			
TUR-P			49 (100)

Anamnestic and clinical data of all enrolled patients and controls. Data in parentheses are percentages. BCG, bacillus Calmette-Guérin; TUR-P, transurethral resection of the prostate.

a Chemotherapy with 30 mg epirubicin.

**Table II:** Follow-up results according to IL-6/IL-10 value.

Group	IL-6/IL-10	Status at follow-up			Total
		VNED	VREC with progression	VREC without progression	
A	≤0.09	15	2	21	38
B	>0.10	24	1	9	34
Total		39	3	30	72

Follow-up results according to IL-6 and IL-10 ratio value. IL-6, interleukin-6; IL-10, interleukin-10. VNED, alive without recurrence; VREC, alive with at least one recurrence.

**Table III:** Multivariate analysis results of factors affecting recurrence risk in all enrolled patients.

Categories (variables)	No. of recurrence-free patients/Total no. of patients (%)	Multivariate analysis HR (95% CI) (p)
Age		0.74
<65 years	23/42 (54.7)	0.77 (0.60–1.43)
≥65 years	16/30 (53.3)	0.62 (0.15–1.16)
Gender		0.71
Male	38/70 (54.2)	1.00 (0.18–1.55)
Female	1/2 (50.0)	1.21 (0.75–1.16)
Number of recurrences/year		0.25
1	5/8 (62.5)	0.99 (0.12–1.67)
2	6/15 (40.0)	0.74 (0.10–1.19)
≥3	28/49 (57.1)	1.15 (0.47–2.00)
Diameter of lesions		0.89
<3 cm	29/44 (65.9)	1.01 (0.15–1.23)
≥3 cm	10/28 (35.7)	2.21 (2.10–3.02)
Grade		0.09
G1	3/8 (37.5)	0.97 (0.12–1.13)
G2	16/26 (61.5)	1.10 (0.23–1.24)
G3	20/38 (52.6)	1.99 (0.78–2.32)
Number of lesions		0.03
1	10/11 (90.9)	0.62 (0.24–1.08)
2	13/25 (52.0)	1.29 (0.61–1.99)
≥3	16/36 (44.4)	4.22 (2.62–3.95)
IL-6 levels		0.62
Normal (≤3.12 pg/ml)	21/37 (56.7)	1.68 (1.50–2.87)
Abnormal	18/35 (51.4)	1.12 (0.20–2.12)
IL-10 levels		0.71
Normal (≤7.8 pg/ml)	16/21 (76.1)	0.87 (0.74–1.09)
Abnormal	23/51 (45.0)	1.09 (0.19–1.20)
IL-6/IL-10		0.002
≤0.09	15/38 (39.4)	0.65 (0.29–1.19)
>0.10	24/34 (70.5)	3.87 (2.81–4.98)

Multivariate analysis results of factors affecting recurrence risk in all enrolled patients. Data in parentheses are percentages. HR, hazard ratio, 95% CI, 95% confidence interval. IL-6, interleukin-6; IL-10, interleukin-10.
